# Enhancing Förster Resonance Energy Transfer (FRET) Efficiency of Titania–Lanthanide Hybrid Upconversion Nanomaterials by Shortening the Donor–Acceptor Distance

**DOI:** 10.3390/nano10102035

**Published:** 2020-10-15

**Authors:** Syue-Liang Lin, Han-Chun Chen, Cheng Allen Chang

**Affiliations:** 1 Department of Biomedical Imaging and Radiological Sciences, National Yang-Ming University, Taipei 112, Taiwan; jack0930991420@hotmail.com; 2 Department of Biotechnology and Laboratory Science in Medicine, National Yang-Ming University, Taipei 112, Taiwan; 3 Department of Biomedical Engineering, National Yang-Ming University, Taipei 112, Taiwan; 4 Biomedical Engineering Research and Development Center, National Yang-Ming University, Taipei 112, Taiwan

**Keywords:** Nd^3+^ sensitized upconversion, lanthanide, photodynamic therapy

## Abstract

Several robust titania (TiO_2_) coated core/multishell trivalent lanthanide (Ln) upconversion nanoparticles (UCNPs) hybrid architecture designs have been reported for use in photodynamic therapy (PDT) against cancer, utilizing the near-infrared (NIR) excited energy down-shifting and up-conversion chain of Nd^3+^ (λ_793-808 nm_) → Yb^3+^ (λ_980 nm_) → Tm^3+^(λ_475 nm_) → TiO_2_ to produce reactive oxygen species (ROS) for deep tissue-penetrating oxidative cytotoxicity, e.g., NaLnF_4_:Yb,Tm (Ln = Y, Gd). Herein, we demonstrate that by doping the Tm^3+^ emitter ions in the outer shell and the Nd^3+^ sensitizer ions in the core, the newly designed NaYF_4_:Nd,Yb@Yb@Yb,Tm@TiO_2_ hybrid UCNPs exert more ROS production than the reference NaYF_4_:Yb,Tm@Yb@Nd,Yb@ TiO_2_ with the Tm^3+^ ions in the core and the Nd^3+^ ions in the outer shell, upon 793 nm laser irradiation, primarily due to the shortening of the Tm^3+^-TiO_2_ distance of the former with greater Förster resonance energy transfer (FRET) efficiency. After coating with polyallylamine hydrochloride (PAH)/polyethylene glycol folate (PEG-FA), the resulting NaYF_4_:Nd,Yb@Yb@Yb,Tm@TiO_2_-PAH-PEG-FA hybrid nanocomposites could be internalized in MDA-MB-231 cancer cells, which also show low dark cytotoxicity and effective photocytotoxicity upon 793 nm excitation. These nanocomposites could be further optimized and are potentially good candidates as nanotheranostics, as well as for other light-conversion applications.

## 1. Introduction

In the precision medicine era, in addition to the current types of cancer treatment, i.e., surgery, radiotherapy, chemotherapy and targeted therapy, several other methods including immunotherapy, [1] phototherapy (i.e., photodynamic and photothermal therapies) [2,3] and stem cell transplants [4] are either in clinical trials or under research development. Among these new developments, photodynamic therapy (PDT) is potentially highly selective, localizable to tumor, less invasive or less of a PDT-induced immune response, and with low side effects as adjuvant therapy for cancer treatment [2,5,6]. In the presence of photosensitizers (PS) and with light excitation, reactive oxygen species (ROS) and/or singlet oxygen (^1^O_2_) molecules are produced for oxidative cytotoxicity [7]. However, conventional photosensitizers mainly absorb UV/Vis light, and the penetration of UV/Vis lights in biological tissues is low—they are only suitable for surface lesions.

Because of the problem of limited tissue penetration depth of UV/Vis lights, near-infrared (NIR) lights with higher biological tissue penetration in the wavelength region of 650–1700 nm have been employed in phototherapy [8,9]. To effectively convert the NIR light to visible light for subsequent energy transfer to conventional PS by the upconversion process, the Nd^3+^/Yb^3+^ co-doped upconversion nanoparticles (UCNPs) [10] were developed, in which the Nd^3+^ and Yb^3+^ ions can be excited at 800 nm [11] and 980 nm [12], respectively.

Due to concurrent down-shifting emissions suitable for imaging applications, these nanomaterials are excellent candidates for diagnostics, and emerging core/shells architecture designs to reduce surface quenching and energy back transfer phenomena have been reported [13,14]. The tissue targeting ability can also be added by derivatization with cancer cell biomarkers such as folate, [15] RGD peptide, [16], and several monoclonal antibodies [17] on the UCNPs with suitable bioconjugate techniques [18].

Recently, the nanocomposites with therapeutic and diagnostic functions for nanotheranostics have enabled patient-tailored combination therapy, drug-release controlling, imaging-guided local therapy, and treatment response monitoring [19]. The development of nanotheranostics is currently under continuous improvements, such as deliver efficiency, long-term toxicity and stability. In this regard, the semiconductor material TiO_2_ (titania) has been chosen as a candidate material. TiO_2_ is well-known as an excellent photocatalyst and has the advantages of high physicochemical stability, low cost, and low cytotoxicity [20,21]. Among the three polymorphic rutile, anatase and brookite forms, under UV excitation, the anatase TiO_2_ can produce relatively more reactive oxygen species (ROS) to kill cancer cells [22]. Coating the TiO_2_ on the surface of the UCNPs NaYF_4_:Yb,Tm allows TiO_2_ to generate ROS upon NIR 980 nm excitation for PDT [23]. Moreover, the addition of a NaGdF_4_:Yb shell between the core NaYF_4_:Yb,Tm and the TiO_2_ layer resulted in enhanced upconverting UV emission, presumably due to suppressed surface quenching, while still maintaining the photocatalytic decomposition activity of methylene blue (MB) [24]. Several other TiO_2_ coated UCNPs have also been reported, including: (i) with dual-TiO_2_/hypocrellin A photosensitizers and hyaluronic acid targeted molecules with 808 nm excitation, [25] (ii) with doxorubicin-loaded NaYF_4_:Yb,Tm-TiO_2_ for chemo- and PDT combination therapy with 980 nm excitation, [26] (iii) with a NaGdF_4_:Yb shell between the core NaGdF_4_:Yb,Tm and the sensitizer NaNdF_4_:Yb and an outer NaGdF_4_ shell (i.e., NaGdF_4_:Yb,Tm@NaGdF_4_:Yb@NaNdF_4_:Yb @NaGdF_4_@mSiO_2_@TiO_2_) to reduce the respective energy back transfer from Tm^3+^ to Nd^3+^ ions and surface quenching [27]. In these nanomaterials, the emitters, Tm^3+^ ions, are constructed in the NaLnF_4_:Yb,Tm (Ln = Y, Gd) core.

It is known that the Förster resonance energy transfer (FRET) efficiency (E) depends on the donor-to-acceptor separation distance r with an inverse 6th-power law due to the dipole–dipole coupling mechanism: E = 1/[1 + (r/R_0_)^6^] with R_0_ being the distance of 50% efficiency (i.e., the Förster distance). Thus, E decays in a sigmoid function fashion with increasing distance between the UCNPs emitters (i.e., Tm^3+^ ions) and photosensitizer acceptors (i.e., TiO_2_) [28,29,30], hence the decreasing amount of ROS production when applied in PDT [31]. If other effects such as surface quenching and energy back transfer of the UCNPs are also considered, the amount of ROS production could be optimized for better overall luminescence energy transfer efficiency and a few examples are known: (i) The core/shell UCNPs NaYF_4_:Yb@Er with spatially inversely distributed sensitizers (Yb) and activators (Er) as compared to the reference NaYF_4_:Er@Yb exerts more luminescence intensity due to shorter distance between the donor-acceptor (e.g., Er-RB, rose bengal) pairs, [32] (ii) a nonemitting outer protective Gd shell was added to result in the core/shell/shell UCNPs NaYbF_4_:Gd@NaYF_4_:Gd,Er@NaYF_4_:Gd and the emission was twice as bright as reference NaYF_4_:Gd,Yb,Er@ NaYF_4_:Gd and up to 8 fold emission enhancement from the surface bound dye chemophores through Er to dye FRET, [33] (iii) the UCNPs NaYF_4_:Nd,Yb@Yb@Yb,Ln (Ln = Er, Tm) with the emitter Er or Tm in the shell and sensitizers Nd,Yb in the core were included for use in photodynamic therapy in our earlier patent application [34]. Note that the above first and second examples were designed by using NIR 980 nm excitation on Yb, which would be considered inferior to the 3rd one by 790–808 nm excitation on Nd as discussed previously.

**Scheme 1:**
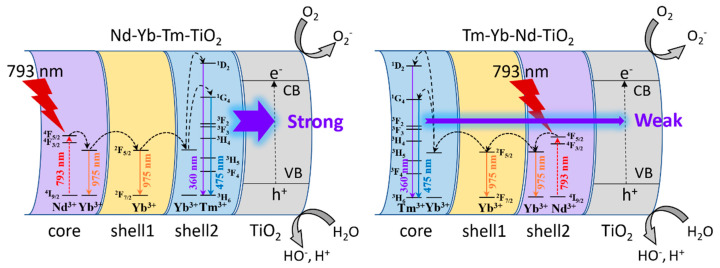
The core/shell/shell hybrid UCNPs Nd-Yb-Tm-TiO_2_ (left) would achieve greater FRET efficiency to produce ROS than the reference Tm-Yb-Nd-TiO_2_ (right), due to shortened Tm^3+^-TiO_2_ distance upon 793 nm NIR laser excitation.

In this study, we demonstrate that by doping the Tm^3+^ emitters in the shell and the Nd^3+^ and Yb^3+^ sensitizers in the core and after the addition of a Yb^3+^ shell and TiO_2_ coating with polyallylamine hydrochloride (PAH)/polyethylene glycol folate (PEG-FA), the newly designed core/shell/shell hybrid UCNPs NaYF_4_:Nd,Yb@Yb@Yb,Tm@TiO_2_-PAH-PEG-FA (designated as Nd-Yb-Tm-TiO_2_-UCNCs) would achieve greater FRET efficiency to produce ROS for PDT as compared to that of the reference NaYF_4_:Yb,Tm@Yb@Nd,Yb@TiO_2_-PAH-PEG-FA (designated as Tm-Yb-Nd-TiO_2_-UCNCs), due primarily to shortened Tm^3+^-TiO_2_ distance upon 793 nm NIR laser excitation (Scheme 1). Note that the excitation at 793 nm on Nd^3+^ doped UCNPs would give about 4 times greater luminescence intensity at 975 nm than that excitation at 808 nm, with lower power and increased safety [35]. To simplify the writing of these UCNPs formulas, the mol% values of the doping Ln^3+^ ions are neglected unless if deemed necessary to clarify certain statements.

## 2. Materials and Methods

### 2.1. Chemicals

Reagent or HPLC grade chemicals were used in this study. All chemicals were used as received without further purification. Yttrium acetate hydrate, ytterbium acetate hydrate, thulium acetate hydrate, neodymium acetate hydrate, oleic acid, 1-octadecene, sodium hydroxide, ammonium fluoride, IGEPAL CO-520, ammonium hydroxide solution, tetraethyl orthosilicate (TEOS), Polyallylamine hydrochloride 17,000 MW, 9,10-Anthracenediyl-bis(methylene) dimalonic acid (ABDA), anatase titanium(IV) oxide, titanium(IV) butoxide (TBOT), *N*-(3-Dimethylaminopropyl)-*N*’-ethylcarbodiimide hydrochloride (EDC-HCl), *N*-Hydroxysulfosuccinimide sodium salt (sulfo-NHS), dimethyl sulfoxide (DMSO), and cell counting kit 8(CCK8) were purchased from Sigma-Aldrich (St Louis, MO, USA); *n*-hexane, acetone, methanol, and ethanol were from Thermo Fisher Scientific (Waltham, MA, USA); PEG-FA-NHS ester (MW 3400) was purchased from Nanocs (New York, NY, USA).

### 2.2. Characterizations

Transmission electron microscope (TEM) Image measurements of nanomaterials were carried out on a JEM-2000EX II (JEOL, Akishima, Tokyo, Japan) at 100 kV. Photoluminescence spectra were measured by FSP-920 (Edinburgh Instruments, Livingston, West-Lothian, Scotland) spectrometer equipped with a 793-nm diode laser. The UV–Vis absorption spectra were measured by Agilent 8453 diode array UV–Vis spectrophotometer (Agilent Technologies, Santa Clara, CA, USA). The dynamic light scattering (DLS) measurement was determined by SZ-100 zetasizer (HORIBA Scientific, Kyoto, Kyoto Prefecture, Japan). Cell imaging was performed on an FV1000-IX81 laser confocal microscope (Olympus, Shinjuku, Tokyo, Japan).

### 2.3. Synthesis of the Core NaYF_4_:Nd(15%),Yb(15%) and NaYF_4_:Yb(10%),Er(0.5%) NPs

The upconversion nanoparticles (UCNPs) were synthesized according to a literature reported method with minor modifications [35]. Core NaYF_4_:Nd,Yb NPs, a total of 1 mmol Ln-triacetate in the Y:Nd:Yb = 70:15:15 mol% ratio (for NaYF_4_:Yb,Tm NPs, in the Y:Yb:Tm = 89.5:10:0.5 mol% ratio) were added to a 10 mL oleic acid (OA) and 15 mL octadecene (ODE) solution. The temperature of solution was increased to 160 °C under argon flow for 60 min. After cooling, 4 mmol NH_4_F and 2.5 mmol NaOH in 10 mL methanol solution containing was added to the solution and stirring for 1 h at about 65 °C. Afterward, the temperature of the solution was increased to 300 °C for 90 min. After cooling, the resulting NaYF_4_:Nd^3+^(15%),Yb^3+^(15%) and NaYF_4_:Yb^3+^ (10%),Tm^3+^(0.5%) NPs were centrifuged for collecting the NPs, the precipitate was washed by ethanol for several times, and redispersed in *n*-hexane for following reaction.

### 2.4. Synthesis of the Core/Shell and Core/Shell/Shell NaYF_4_:Nd(15%),Yb(15%)@NaYF_4_:Yb(20%)@ NaYF_4_:Yb(20%), Tm (0.5%) and NaYF_4_:Yb(10%),Tm(0.5%)@NaYF_4_: Yb(20%)@NaYF_4_:Nd(20%),Yb(10%)

0.4 mmol Y-triacetate and 0.1 mmol Yb-triacetate were added to 10 mL OA and 15 mL ODE solution with stirring. The temperature of solution was increased to 160 °C for 60 min to obtain a homogeneous solution. After the solution was cooled, the synthesized core NaYF_4_:Nd,Yb or NaYF_4_:Yb,Tm NPs in *n*-hexane was added along with 0.05 g NH_4_F and 0.075 g NaOH in 5 mL methanol solution. The solution was stirred for 60 min at 65 °C before heating to 300 °C for 90 min, and then cooled to room temperature. The product core-shell NaYF_4_:Nd^3+^ (15%),Yb^3+^(15%)@NaYF_4_: Yb^3+^(20%) and NaYF_4_:Yb^3+^(10%),Tm^3+^(0.5%)@ Yb^3+^(20%) were collected by centrifugation, washed with ethanol, and redispersed in *n*-hexane. For the process of synthesizing core/shell/shell UCNP, 0.375 mmol Y-triacetate, 0.1 mmol Yb-triacetate and 0.025 mmol Tm-triacetate (or 0.35 mmol Y-triacetate, 0.05 mmol Yb-triacetate and 0.1 mmol Nd-triacetate) were added to 10 mL OA and 15 mL ODE mixed solution. The solution was heated to 180 °C for 60 min to obtain a homogeneous solution. After the solution was cooling, the synthesized core/shell NaYF_4_:Nd,Yb@NaYF_4_:Yb or NaYF_4_:Yb,Tm@ NaYF_4_:Yb NPs in *n*-hexane was added along with 0.05 g NH_4_F and 0.075 g NaOH in 5 mL methanol solution. The mixture was heated to 100 °C to evaporate some organic solvent before heating to 300 °C for 90 min. The solution was cooled to room temperature and the resulting core/shell/shell UCNPs NaYF_4_:Nd^3+^(15%),Yb^3+^ (15%)@NaYF_4_: Yb^3+^(20%)@NaYF_4_:Yb^3+^(20%),Tm^3+^(0.5%) and NaYF_4_:Yb^3+^(10%),Tm^3+^(0.5%)@Yb^3+^(20%)@Nd^3+^(20%),Yb^3+^ (10%) were centrifuged, washed with ethanol several times, and redispersed in *n*-hexane.

In an alternative method, the multishell nanoparticles were prepared according to the literature reported method using the Successive Layer-by-Layer Strategy (SLBL) with some modifications [36]. The preparation of the core/shell precursors was performed first: 2.5 mmol Ln(CH_3_COO)_3_ (Ln = Y, Yb, Ln = Y, Nd, Yb for core/shell/shell) was added to a solution containing 10 mL OA and 15 mL ODE with stirring. The mixture was then heated to 180 °C for 1 h to obtain a 0.1 M Ln-OA homogeneous precursor solution, which was then cooled to room temperature. In the meantime, a 0.4 M CF_3_COONa (Na-TFA) stock solution in 10 mL OA was prepared under vacuum with stirring to remove residual oxygen and water. Then, 3 mL of the previously synthesized core NaYF_4_:Nd,Yb or NaYF_4_:Yb,Tm NPs in *n*-hexane was added to a solution containing 6 mL OA and 15 mL ODE in a flask. The solution was heated to 65 °C to evaporate *n*-hexane before heating to 290 °C under an argon flow. Then, 1 mL 0.1 M Yb-OA and 0.5 mL 0.4 M Na-TFA-OA stock solutions were added sequentially at 290 °C, at a 10 min duration time. After cooling to room temperature, the core/shell UCNPs NaYF_4_:Nd,Yb@ NaYF_4_: Yb or NaYF_4_:Yb,Tm@NaYF_4_:Yb was centrifuged, washed with ethanol several times, and redispersed in 15 mL *n*-hexane. The core/shell/shell UCNPs can then be prepared according to similar procedures. The amounts of the added lanthanide salt stock solutions could be adjusted according to the molar ratio required.

### 2.5. Coating TiO_2_ on the Core/Shell/Shell UCNPs

A reverse water-in-oil microemulsion method was used. To 1 mL of the surfactant Igepal CO-520 dispersed in 25 mL *n*-hexane was added 5 mg oleic acid capped core/shell/shell UCNPs in 1 mL hexane solution. After mixing for 30 min, 140 mL ammonia hydroxide solution was added gently, and the solution was ultrasonicated to form spherical droplets in the nonpolar solvent [37]. Then 80 mL tetraethyl orthosilicate (TEOS) was slowly added into the solution and the mixture was kept stirring for 24 h. The core/shell/shell UCNPs@dSiO_2_ products were centrifuged, washed with ethanol several times to remove excess reagents. The solids were redispersed in 25 mL ethanol for further TiO_2_ coating, which was added 0.06 mL ammonia hydroxide solution and stirred for 30 min. The solution was heated and kept at 45 °C and 0.2 mL of titanium(IV) butoxide (TBOT) was added dropwise, and the mixture was stirred for 12 h and cooled to room temperature. The UCNPs coated with TiO_2_ (UCNPs@dSiO_2_@TiO_2_) were separated by centrifugation and washed several times with ethanol, and transferred into 25 mL ddH_2_O, sealed in a glass container, and allowed for sintering in an autoclave at 180 °C for 6 h to convert the phase of TiO_2_ from rutile to anatase [24]. After the container was cooled to room temperature, the products (i.e., UCNPs@TiO_2_) were separated by centrifugation and washed with ethanol and deionized water several times and stored in ethanol.

### 2.6. Preparation of UCNP@TiO_2_-PAH-PEG-FA

The amino-functionalized UCNPs@TiO_2_-PAH was obtained by mixing 5 mg of UCNPs@TiO_2_ in 10 mL ethanol and 100 μL (20 wt %) PAH and the mixture was stirred for 12 h at room temperature. After centrifugation, the UCNPs@TiO_2_-PAH product was collected and dispersed in 15 mL ddH_2_O, which was further reacted with 5 mg FA-PEG-NHS [18] and stirred for 12 h at room temperature to obtain UCNPs@TiO_2_-PAH-PEG-FA. The final product was separated by centrifugation, washed and redispersed in ddH_2_O and stored in 4 °C for further use. The size stability of UCNPs@TiO_2_-PAH-PEG-FA was tested by measuring the DLS diameters in solution on day 1 and day 7 after preparation.

### 2.7. Reactive Oxygen Species Determination under 793 nm NIR Laser Irradiation

In a typical experiment, [38] 10 μM ABDA−DMSO solution was added to 2 mL of UCNPs@TiO_2_-PAH-PEG-FA aqueous solution. The mixture was then irradiated using a 793 nm NIR laser with 1 W/cm^2^, and the fluorescence intensities of ABDA at 407 nm were measured in triplicates to correlate with the amounts of ROS generated. The photostability of UCNPs@TiO_2_-PAH-PEG-FA in solution was tested by the ROS produced after standing for 1 day and 7 days in a similar procedure.

### 2.8. Cytotoxicity Assays

The MDA-MB-231 cell line was obtained from Food Industry Research and Development Institute (Hsinchu City, Taiwan). The cells were seeded in 96-well plates (1 × 10^4^ cells/well). After incubation in Dulbecco’s Modified Eagle Medium (DMEM) for 24 h at 37 °C under 5% CO_2_, 100 μL solutions of the UCNPs@TiO_2_-PAH-PEG-FA in the concentration range of 50–800 μg/mL were added into each well and incubated for 24 h and washed by PBS buffer. Then, 50 μL CCK-8 reagent (10 times dilution with DMEM) was added into each well. After incubation for 1.5 h at 37 °C, the cell viability was determined by measuring the absorbance at 450 nm in each well. The data were shown as mean ± SD (n = 3).

### 2.9. In Vitro NIR Induced PDT Effect

MDA-MB-231 cancer cells were seeded in 96-well plates (1 × 10^4^ cells/well) and incubated in DMEM for 24 h at 37 °C under 5% CO_2_. Then, 200 µL of the UCNPs@TiO_2_-PAH-PEG-FA (400 μg/mL) was added into each well and incubated for another 24 h. The treated cells (with UCNPs@TiO_2_-PAH-PEG-FA) and the control group cells (without UCNPs@TiO_2_-PAH-PEG-FA) were irradiated with a 793 nm laser (1–4 W/cm^2^) for 30 min, followed by incubation for another 24 h and addition of the CCK-8 reagent (50 μL, 10 times dilution with DMEM) to each well. After incubation for 1.5 h at 37 °C, the cell viability was determined by measuring the absorbance at 450 nm in each well. The data were shown as mean ± SD (n = 3).

### 2.10. In Vitro Cellular Imaging

MDA-MB-231 cancer cells were seeded in six-well culture dishes at a concentration of 5 × 10^5^ cells/well (2 mL) and incubated in DMEM for 24 h at 37 °C under 5% CO_2_ with and without the addition of the UCNPs@TiO_2_-PAH-PEG-FA (300 μg). All the cells were then incubated for 4 h and washed with PBS buffer solution to fully remove any excess UCNC-FAs. The cells were fixed by adding para-formaldehyde (2 wt%, 1 mL) in each culture dish for 10 min and the cell nuclei were stained with Hoechst 33342 for 10 min. After washing with PBS solution, the cells were imaged using a laser confocal microscope FV1000 (Olympus, Shinjuku, Tokyo, Japan).

## 3. Results and Discussion

### 3.1. Design, Syntheses, Morphology and Luminescence Characterizations of the New Core/Shell/Shell UCNPs NaYF_4_:Nd^3+^(15%),Yb^3+^(15%)@NaYF_4_:Yb^3+^(20%)@NaYF_4_:Yb^3+^(20%),Tm^3+^(0.5%), i.e., Nd-Yb-Tm-UCNPs

**Figure 1:**
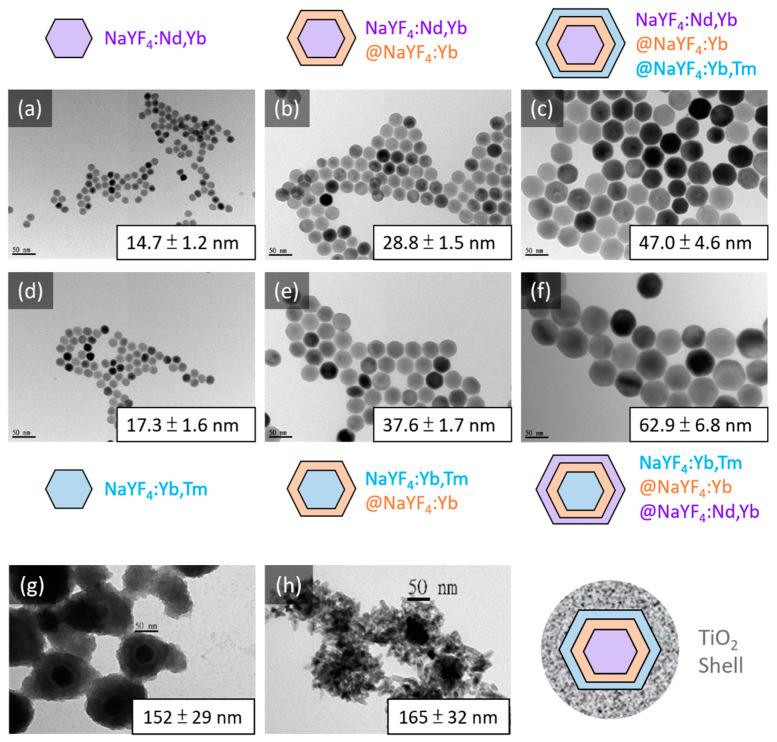
TEM images and DLS analyses data of the core, core/shell and core/shell/shell UCNPs and derivatives. (**a**–**c**) NaYF_4_:Nd,Yb@Yb@Yb,Tm; (**d**–**f**) the reference NaYF_4_:Yb,Tm@Yb@Nd,Yb. (**g**) Nd-Yb-Tm-UCNPs@dSiO_2_@TiO_2_; (**h**) Nd-Yb-Tm-UCNPs@TiO_2_. The particle size determined by DLS analysis is shown on the bottom of each image.

The new design uses NIR 793 nm laser to excite the Nd^3+^ ions and through the energy down-shifting and up-conversion chain of Nd^3+^(λ_793-808 nm_) → Yb^3+^(λ_980 nm_) → Tm^3+^(λ_475 nm_) → TiO_2_ to produce ROS. The NaYF_4_:Yb,Tm emitter shell is on the outside of the sensitizers core NaYF_4_:Yb,Nd to shorten the Tm^3+^-TiO_2_ distance for better FRET efficiency. A NaYF_4_:Yb shell is added in between to reduce the energy back transfer from Tm^3+^ to Nd^3+^ ions [13]. The syntheses of the UCNPs were performed by a thermal decomposition method with the successive layer-by-layer (SLBL) strategy to ensure that the various shells could be uniformly coated on the surfaces of the cores [36]. The doped Tm^3+^ concentration was the previously optimized 0.5 mol% for best emission at 475 nm [39]. The doped Yb^3+^ concentrations were the partially optimized 10–20 mol% for greater upconversion efficiency, [40] and concentration quenching occurred beyond 20 mol% [41]. The transmission electron microscope (TEM) images and dynamic light scattering (DLS) analyses data of the core, core/shell and core/shell/shell UCNPs Nd-Yb-Tm-UCNPs show that they are uniform and well-dispersed (Figure 1a–c). The DLS diameters increase with increasing number of coated shells: from core 14.7 nm to core/shell 28.8 nm and core/shell/shell 47.0 nm. The combined shell thickness is 16.2 nm.

**Figure 2:**
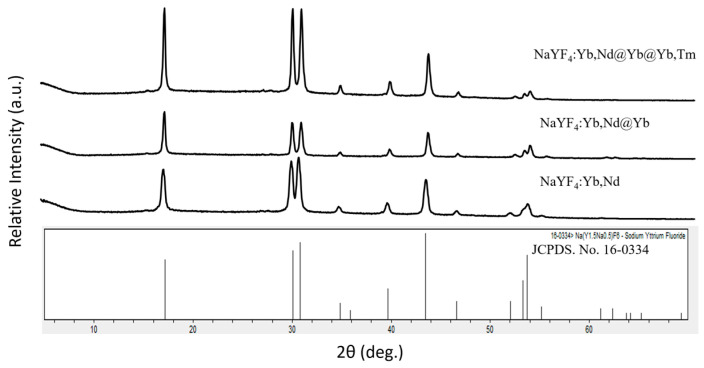
XRD patterns of the core, core/shell and core/shell/shell UCNPs NaYF_4_:Nd,Yb@Yb@Yb,Tm, in comparison with Joint Committee on Powder Diffraction Standards (JCPDS). No. 16-0334 β-NaYF_4_.

**Figure 3:**
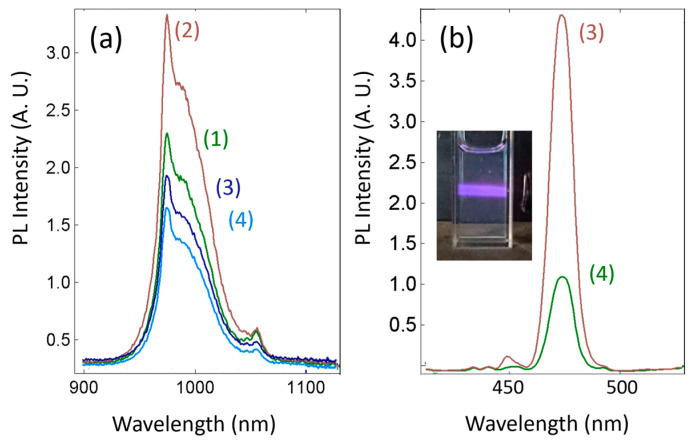
(**a**) Downshifting emission spectra of NaYF_4_:Nd,Yb (1), NaYF_4_:Nd,Yb@Yb (2), NaYF_4_:Nd,Yb@Yb@Yb,Tm (3) and reference NaYF_4_:Yb,Tm@Yb@Nd,Yb (4) upon 793 nm excitation; (**b**) upconversion emission spectra of NaYF_4_:Nd,Yb@Yb@Yb,Tm (3) and the reference NaYF_4_:Yb,Tm@Yb@Nd,Yb (4) with 793 nm excitation, Inset: photograph of NaYF_4_:Nd,Yb@Yb@Yb,Tm in *n*-hexane upon 793 nm excitation.

For comparison purpose, the reference UCNPs NaYF_4_:Yb^3+^(10%),Tm^3+^(0.5%)@Yb^3+^(20%)@Nd^3+^(20%),Yb^3+^(10%) (i.e., Tm-Yb-Nd-UCNPs) with the Tm emitter in the core and the Nd sensitizer in the outer shell were also synthesized and studied (Figure 1d–f). The DLS diameters also increase with increasing number of coated shells: from core 17.3 nm to core/shell 37.6 nm and to core/shell/shell 62.9 nm. The combined shell thickness is estimated to be 22.8 nm, which is also the Tm^3+^-TiO_2_ distance after TiO_2_ coating (vide infra). The respective X-ray diffraction (XRD) patterns and EDS data confirm that these UCNPs are hexagonal (Figure 2) and the surface elemental contents of selected nanomaterials, i.e., core and core/shell/shell (data not shown). Concerning the luminescence properties, it is observed that after the addition of a NaYF_4_:Yb shell, the down-shifting 980 nm luminescence intensity of the resulting NaYF_4_:Nd,Yb@Yb increases to 1.5 times of that of NaYF_4_:Nd,Yb (λex = 793 nm), presumably due to partially suppressed surface quenching (Figure 3a). Addition of the next NaYF_4_@Yb,Tm shell reduces the 980 nm luminescence intensity of the resulting UCNPs NaYF_4_:Nd,Yb@Yb@Yb,Tm due to upconversion energy from Yb^3+^ to Tm^3+^ ions (λex = 793 nm). Note that this 980 nm luminescence intensity is slightly greater than that of the reference NaYF_4_:Yb,Tm@Yb@Nd,Yb (Figure 3a, light blue) because the latter has greater Nd → Yb surface quenching.

The characteristic Tm^3+^ ion upconversion emission spectra with clearly detectable peaks at 345 nm (^1^I_6_ → ^3^F_4_), 360 nm (^1^D_2_ → ^3^H_6_), 450 nm (^1^D_2_ → ^3^F_4_) and 475 nm (^1^G_4_ → ^3^H_6_) for both UCNPs NaYF_4_:Nd,Yb@Yb@Yb,Tm and the reference NaYF_4_:Yb,Tm@Yb@Nd,Yb could be measured upon excitation at 793 nm (Figure 3b). The highest 475 nm luminescence intensity is lower for NaYF_4_:Nd,Yb@Yb@Yb,Tm as compared to that of the reference NaYF_4_:Yb,Tm@Yb@Nd,Yb because the latter has less Yb→Tm surface quenching. A similar trend was observed for the upconversion 475 nm luminescence intensities upon 980 nm excitation. Both are greater than those of the core/shell NaYF_4_:Yb(40%),Tm@NaGdF_4_:Yb and core only NaYF_4_:Yb^3+^(20%),Tm UCNPs (data not shown). The reasons for the observed trend could be related to the interplay of whether the UCNPs consist one or more shielding shells to suppress surface quenching and the fine-tunning of relative concentrations of the upconversion Yb^3+^ ion sensitizer in the Tm shell [42]. Note that the 475 nm luminescence intensity of the 30 nm UCNPs NaGdF_4_:Yb,Tm@Gd with a protective NaGdF_4_ shell upon 980 nm excitation is 60 times that of NaGdF_4_:Yb,Tm due to suppressed surface quenching [43].

### 3.2. TiO_2_ and PAH/PEG-FA Modifications and Zeta Potential Changes

The new core/shell/shell Nd-Yb-Tm-UCNPs were prepared and collected in the forms of oleic acid adsorbed hydrophobic nanomaterials. In order to add a TiO_2_ coated layer with a tetragonal structure onto these hexagonal materials, the dense-silica (dSiO_2_) coated hydrophilic intermediate template materials (designated as Nd-Yb-Tm-UCNPs@dSiO_2_) through the Si-O-Ln and Si-O-Si bond formations were prepared by adding tetraethyl orthosilicate (TEOS) via hydrolysis and condensation reactions. The titanium(IV) butoxide (TBOT) was then added to form Nd-Yb-Tm-UCNPs@dSiO_2_@TiO_2_ through the Si-O-Ti bond formation [37]. The final mesoporous anatase TiO_2_ coated hybrid Nd-Yb-Tm-UCNPs@TiO_2_ (i.e., Nd-Yb-Tm-TiO_2_) was obtained by sintering Nd-Yb-Tm-UCNPs@dSiO_2_@TiO_2_ at 180 °C in an autoclave. The DLS diameter of the aggregated intermediate TiO_2_ coated Nd-Yb-Tm-UCNPs@dSiO_2_@TiO_2_ is 152 nm with a ~20 nm dSiO_2_/TiO_2_ thickness (Figure 1g). The DLS diameter of the final mesoporous Nd-Yb-Tm-TiO_2_ is 165 nm (Figure 1h), which has a strong absorption band in the 280–380 nm range.

The Nd-Yb-Tm-TiO_2_ hybrid UCNPs were subjected to ROS production tests together with the reference Tm-Yb-Nd-TiO_2_ and negative controls, before further coating with polyallylamine hydrochloride (PAH)/polyethylene glycol folate (PEG-FA) to obtain Nd-Yb-Tm-UCNPs@TiO_2_-PAH-PEG-FA (designated as Nd-Yb-Tm-TiO_2_-UCNCs) for subsequent stability, imaging and PDT cytotoxicity studies. The respective DLS diameters for the Nd-Yb-Tm-UCNPs@TiO_2_-PAH and Nd-Yb-Tm-TiO_2_-UCNCs are 202 nm and 227 nm. The zeta potentials for the various selected surface-modified UCNPs were measured to be: Nd-Yb-Tm-UCNPs@dSiO_2,_ −40.8 mV; Nd-Yb-Tm-UCNPs@dSiO_2_@TiO_2,_ −47.0 mV; Nd-Yb-Tm-UCNPs@TiO_2_ -PAH, +61.8 mV; and Nd-Yb-Tm-TiO_2_-UCNCs, +14.8 mV. These results are consistent with the nature of their surface modifications, i.e., negative zeta potentials for SiO_2_ and TiO_2_ coated nanomaterials and positive zeta potentials for PAH and PEG-FA modified nanomaterials with residual positively charged -NH_2_ functional groups.

### 3.3. ROS Production, Stability, Imaging and PDT Cytotoxicity Studies

**Figure 4:**
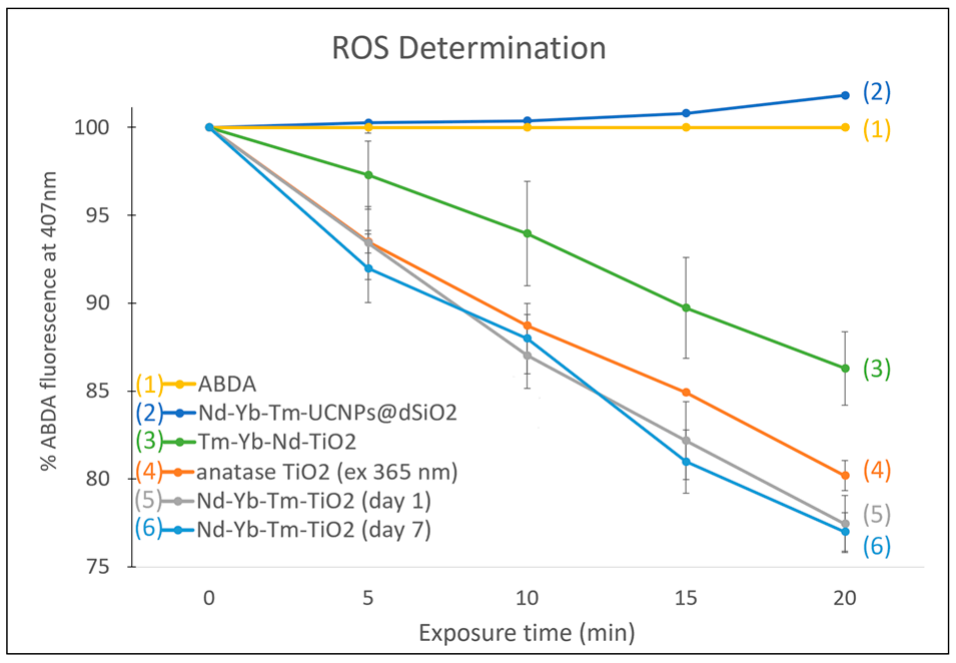
ROS production by 793 nm irradiations (1 W/cm^2^) on Nd-Yb-Tm-TiO_2_(5,6) with different control and reference materials: ABDA only(1), Nd-Yb-Tm-UCNPs@dSiO_2_(2), reference Tm-Yb-Nd-TiO_2_(3) and anatase TiO_2_ (4, λex 365 nm); Conc. 1 mg/mL.

The 9,10-Anthracenediyl-bis(methylene)dimalonic acid (ABDA) was used to determine the generated reactive oxygen species (ROS) after 793 nm NIR laser irradiation on the TiO_2_-coated UCNPs. Irradiations of the Nd-Yb-Tm-TiO_2_ with different control and reference materials by 793 nm NIR laser (1 W/cm^2^) for up to 20 min in the presence of ABDA allow the determinations of ROS production, and the results are shown in Figure 4.

It is observed that ROS production increases with increasing irradiation time. The newly designed Nd-Yb-Tm-TiO_2_ after 20 min 793 nm laser irradiation results in 23% reduction of ABDA luminescence at 407 nm due to ROS production which is greater than that (i.e., 14%) of the reference Tm-Yb-Nd-TiO_2_ and that (i.e., 20%) of anatase TiO_2_ upon 20 min 365 nm irradiation. The greater ROS production of Nd-Yb-Tm-TiO_2_ is attributed to the shorter Tm^3+^-TiO_2_ distance as compared to that of Tm-Yb-Nd-TiO_2_ (i.e., 22.8 nm, vide supra), which leads to greater FRET efficiency. As expected, ABDA and Nd-Yb-Tm-UCNPs@dSiO_2_ (without TiO_2_) do not produce ROS.

**Figure 5:**
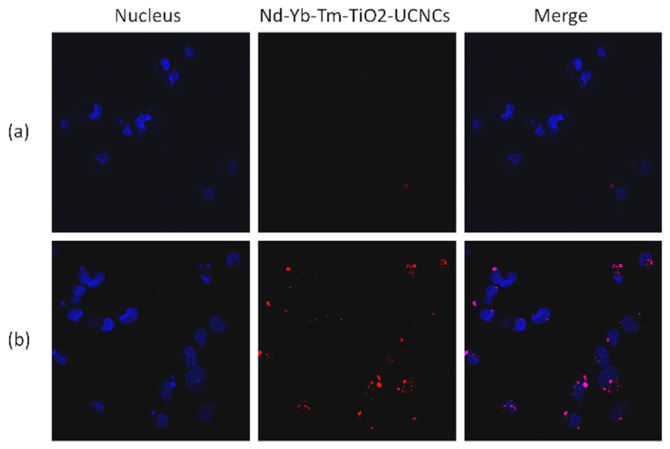
Confocal cell images of MDA-MB-231 cells incubation (**a**) without and (**b**) with the addition of Nd-Yb-Tm-TiO_2_-UCNCs (0.3mg/mL), 37 °C.

Initial stability test of the Nd-Yb-Tm-UCNPs@dSiO_2_, Nd-Yb-Tm-UCNPs@TiO_2_ and Nd-Yb-Tm-TiO_2_-UCNCs indicates that their sizes measured by DLS do not change within 7 days. Unlike the organic photosensitizers rose bengal, methylene blue and hematoporphyrin, repeated uses of the Nd-Yb-Tm-TiO_2_-UCNCs on day 1 and day 7 give very similar ROS productions (Figure 4). In the meantime, the cell uptake and laser confocal imaging studies have been performed using the MDA-MB-231 cancer cells and Nd-Yb-Tm-TiO_2_-UCNCs with a known routine procedure (Figure 5). It is clearly seen that the Nd-Yb-Tm-TiO_2_-UCNCs materials were internalized in the cells through endocytosis. Note that due to the limitation of the equipment, UV light at 395 nm instead of NIR 793 nm was used to perform the cell imaging studies. On the other hand, the Nd→Yb down-shifting 980 nm luminescence of these materials could be easily applied for NIR tumor imaging studies similar to what we published using homemade imaging equipment the presence of an 850-nm longpass (Schott RG-850, 50.8 mm Sq.) filter and using a published experimental procedure [44].

**Figure 6:**
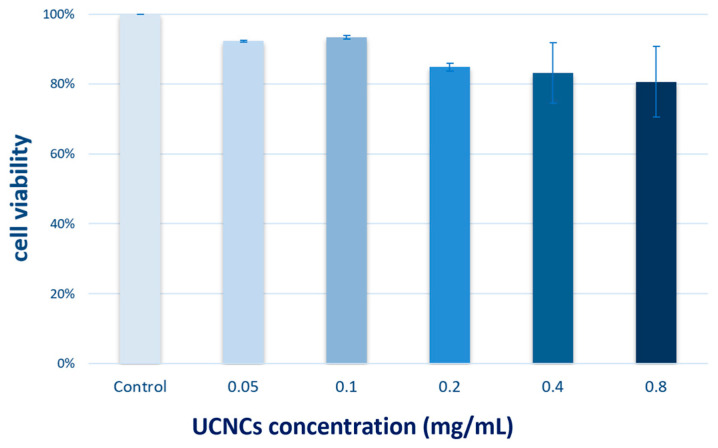
Photodynamic cytotoxicity studies of the Nd-Yb-Tm-TiO_2_-UCNCs (i.e., NP) using MDA-MB-231 cancer cells, at 37 °C, after 24 h incubation.

Cytotoxicity of the UCNPs@TiO_2_-PAH-PEG-FA was evaluated by employing MDA-MB-231 cancer cells. The photodynamic cytotoxicities of the Nd-Yb-Tm-TiO_2_-UCNCs have been further tested against MDA-MB-231 cancer cells at the dosage range of 0–0.8 mg/mL, with and without 793 nm laser irradiation. It is observed that the dark cell viability (i.e., without 793 nm irradiation) is still more than 80% at a dose of 0.8 mg/mL (Figure 6).

**Figure 7:**
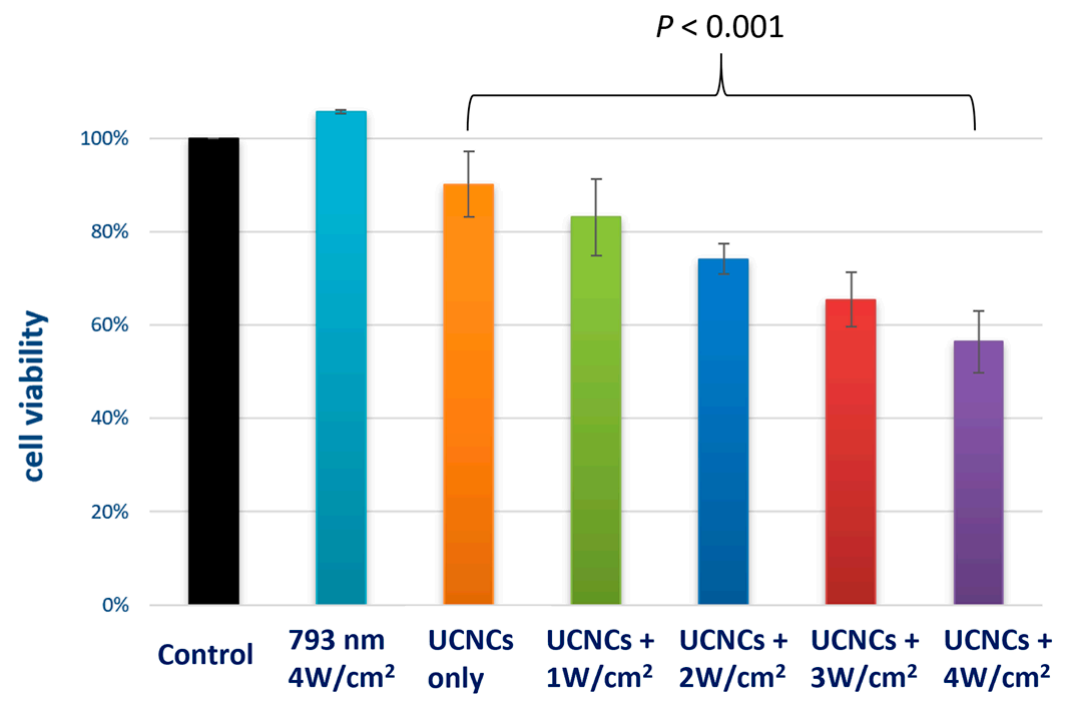
Photocytotoxicities with 793 nm irradiation (1–4 W/cm^2^, 30 min) of MDA-MB-231 cells and 0.4 mg/mL NP.

ROS induced apoptosis was also shown to occur at all phases of cell-cycle [45]. A previously published paper using the material NaYF_4_:Yb,Tm@Yb@TiO_2_, which was similar to our reference material NaYF_4_:Yb,Tm@Yb@Yb,Nd@TiO_2_ has demonstrated in real time the intracellular ROS generation analyses that there was a positive correlation between the amount of ROS and duration time of light irradiation [23]. Our photocytotoxicity study results at a dosage of 0.4 mg/mL Nd-Yb-Tm-TiO_2_-UCNCs and MDA-MB-231 cancer cells with 793 nm irradiation (1–4 W/cm^2^, 30 min) are shown in Figure 7. It is observed that the cell viability decreases with increasing 793 nm irradiation power and reached 54% at 4 W/cm^2^, which are consistent with the results of ROS production study (Figure 4, vide supra) and comparable to the results of others [25]. Note that for cytotoxicity studies, up to 6 W/cm^2^ power has been reported [27]. Certainly, for future in vivo studies, a lower laser power should be used. Recently, the propensity of glutathione (GSH), scavenging ROS radicals, to protect DNA against ROS-induced DNA damage has been studied [46]. It should be noted that the concentration of GSH upregulated in some cancer cells, which may limit the effectiveness of PDT-based therapeutics. Thus, this issue should be considered in related research in the future.

## 4. Conclusions

In summary, by doping the Nd^3+^ sensitizer in the core and the Tm^3+^ emitter in the outer shell to shorten the Tm^3+^-TiO_2_ distance, the newly designed TiO_2_ coated core/shell/shell UCNPs NaYF_4_:Nd,Yb@Yb@Yb,Tm@TiO_2_ exert more ROS production than the reference UCNPs NaYF_4_:Yb,Tm@Yb@Nd,Yb@TiO_2_ with the Tm^3+^ emitter in the core and the Nd^3+^ sensitizer in the outer shell upon 793 nm laser irradiation, presumably due to greater Förster resonance energy transfer (FRET) efficiency. A middle NaYF_4_:Yb^3+^ shell was also added to reduce energy back transfer from Tm^3+^ to Nd^3+^ ions. After coating with PAH-PEG-FA, the resulting TiO_2_ and UCNPs hybrid nanocomposites could be internalized in MDA-MB-231 cancer cells for confocal cell imaging and show low dark cytotoxicity. At a dose of 0.4 mg/mL of the hybrid nanocomposites and upon 793 nm (4 W/cm^2^) irradiation for 30 min, the cell viability reduces to 54% indicating good PDT effect. The initial 7-day stability studies show that they are rather robust as compared to those with organic photosensitizers. In a recent study of ours, [44] it was implied that maximum luminescence should be observed with minimum surface quenching and Tm^3+^ to Nd^3+^ energy back transfer. Because the FRET process is inversely proportional to the sixth power of the distance between the Tm emitter and the TiO_2_ photosensitizer, an optimised ROS production could be obtained with a compromised Tm-TiO_2_ distance. Further exciting studies of these newly designed, potential nanotheranostics are underway including the development of more suitable TiO_2_ coating procedures, fine-tunning of the doping concentrations of trivalent lanthanide sensitizers, activators and emitters to optimize the hybrid UCNPs for better FRET, safety and efficacy for PDT, as well as for other applications such as drug release control and solar energy conversion.
